# Geographic and Genomic Distribution of SARS-CoV-2 Mutations

**DOI:** 10.3389/fmicb.2020.01800

**Published:** 2020-07-22

**Authors:** Daniele Mercatelli, Federico M. Giorgi

**Affiliations:** Department of Pharmacy and Biotechnology, University of Bologna, Bologna, Italy

**Keywords:** SARS-CoV-2, genome evolution, COVID-19, genomics, coronavirus

## Abstract

The novel respiratory disease COVID-19 has reached the status of worldwide pandemic and large efforts are currently being undertaken in molecularly characterizing the virus causing it, SARS-CoV-2. The genomic variability of SARS-CoV-2 specimens scattered across the globe can underly geographically specific etiological effects. In the present study, we gather the 48,635 SARS-CoV-2 complete genomes currently available thanks to the collection endeavor of the GISAID consortium and thousands of contributing laboratories. We analyzed and annotated all SARS-CoV-2 mutations compared with the reference Wuhan genome NC_045512.2, observing an average of 7.23 mutations per sample. Our analysis shows the prevalence of single nucleotide transitions as the major mutational type across the world. There exist at least three clades characterized by geographic and genomic specificity. In particular, clade G, prevalent in Europe, carries a D614G mutation in the Spike protein, which is responsible for the initial interaction of the virus with the host human cell. Our analysis may facilitate custom-designed antiviral strategies based on the molecular specificities of SARS-CoV-2 in different patients and geographical locations.

## Introduction

Initially reported in mid-December 2019 in the Chinese city of Wuhan, the newly emerged severe acute respiratory syndrome virus (SARS-CoV-2) is a single-stranded RNA beta-coronavirus with a compact 29,903 nucleotides-long genome. This virus causes a serious disease known as Coronavirus Disease 2019 (COVID-19), which has spread in over 210 countries in <4 months, counting more than 10 million confirmed cases and almost 500,000 deaths reported worldwide as of June 28, 2020 (source: World Health Organization). A difference in case fatality rates across countries was observed, possibly due to a diverse demographic composition and the type of measures that have been taken in different countries to limit viral spreading (Dowd et al., 2020). According to data from the public database of the Global Initiative on Sharing All Influenza Data (GISAID), three major clades of SARS-CoV-2 can be identified (Forster et al., 2020), that have been subsequently named as clade G (variant of the spike protein S-D614G), clade V (variant of the ORF3a coding protein NS3-G251), and clade S (variant ORF8-L84S). However, as more complete sequences become available, the need to define specific geographic distributions of virus variants becomes of practical importance to define clinical and political strategies at the local level. Despite several reports having confirmed a relatively low variability of SARS-CoV-2 genomes (Ceraolo and Giorgi, 2020; Lu et al., 2020), it is still unclear if different fatality rates or speed of transmission observed in different countries may be the consequence of clade's differences in virulence, as discussed by a recent commentary comparing different strains in the USA (Brufsky, 2020). It is therefore possible that more insights into the pathogenesis and virulence of this virus may come from comparative genomic analysis linked to epidemiologic data coming from different countries.

Genetic variance analyses must now play a crucial role in expanding knowledge on this new virus to adopt measures to contain its outbreak. Complete viral genome sequences have been made rapidly publicly available to the research community and have recently surpassed the 48,000 units, thanks to the worldwide effort of scientists and to the GISAID consortium. This data avalanche will result in an unprecedently rapid effort to analyze data to understand genome diversity (Andersen et al., 2020; Shen et al., 2020), to hypothesize suitable targets for drug repositioning (Wu et al., 2020; Zhou et al., 2020) and to develop prevention strategies (Zhao and Chen, 2020). In the present study, we performed the largest comparative study so far by analyzing more than 48,000 complete SARS-CoV-2 genomes. We report all mutations and stratify them genomically and geographically, also highlighting insurgence of sub-clades and genomic highly variable spots. These finding may be extremely useful to design and think about the efficacy of measures that have been taken on a regional basis to limit SARS-CoV-2 spreading.

## Methods

Forty-eight thousand six hundred thirty-five SARS-CoV-2 genomic sequences were downloaded from GISAID (Shu and McCauley, 2017) on June 26, 2020 (Supplementary File 1). Only viruses affecting human hosts were selected, removing low-quality sequences (>5% NNNs) and using only full-length sequences (>29,000 nt). Forty-eight thousand six hundred twenty-four sequences were associated to a geographic region, specifically: 514 from Africa, 3,340 from Asia, 31,818 from Europe, 10,250 from North America, 2,127 from Oceania and 575 from South America. Eleven sequences were not associated to any continent. We provide as Supplementary File 2 a full geographic description of each sample used in the study.

The reference NC_045512.2 SARS-CoV-2 Wuhan genome (Coronaviridae Study Group of the International Committee on Taxonomy of Viruses, 2020), 29,903 nucleotides long, was obtained from NCBI GenBank. A GFF3 annotation associated to the refence, showing genomic coordinates for all protein sequences of SARS-CoV-2, is provided as Supplementary File 3. The large ORF1 polyprotein was split into its constituent Non-structural proteins (NSPs). The NSP12, encoding for the viral RNA-dependent RNA polymerase, was considered in the annotation as two regions, NSP12a and NSP12b, corresponding to the regions before and after a ribosomal frameshift, occurring as nucleotide 13,468 is translated as both the last nucleotide of a codon and the first of the next codon.

NUCMER version 3.1 (Delcher, 2002) was used to align all 48,635 genome sequences over the NC_045512.2 reference. The output of the alignment was converted to an annotated list of all mutational events using an internally developed R SARS-CoV-2 annotation algorithm provided as Supplementary File 4.

SARS-CoV-2 5′UTR RNA secondary structure has been predicted by free energy minimization together with equilibrium partition function and base pair binding probabilities algorithm from the RNAfold WebServer using default settings (Gruber et al., 2008).

## Results

**Figure 1:**
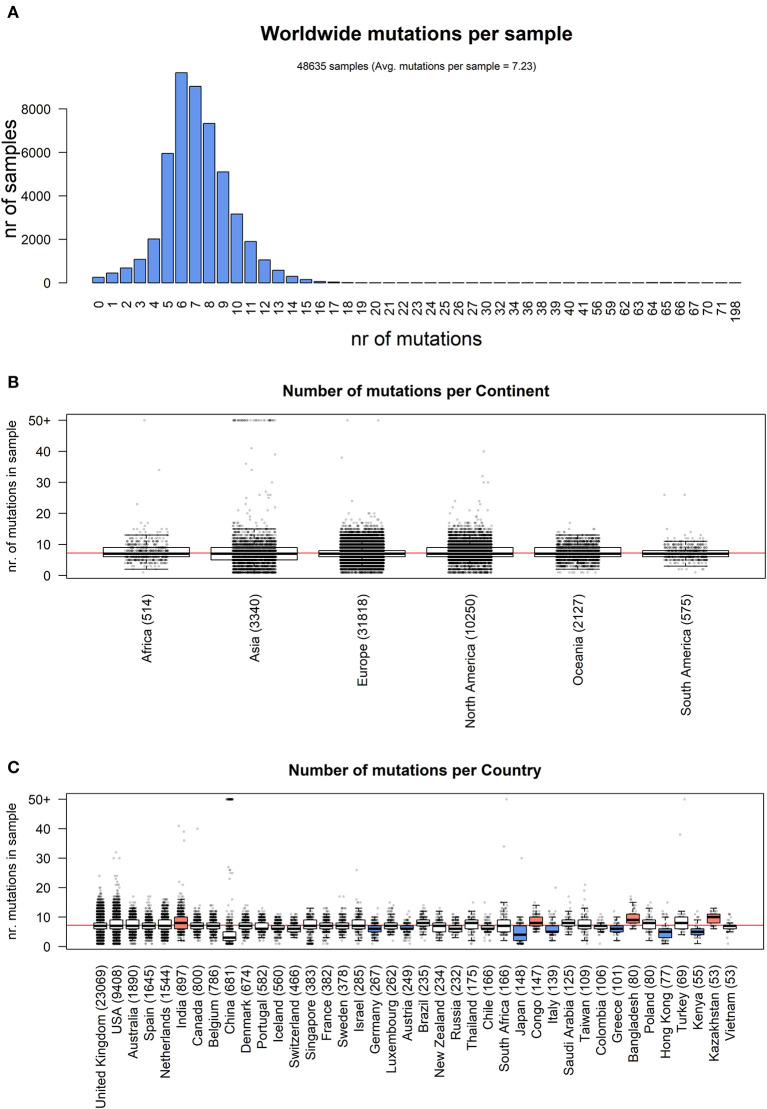
**(A)** Distribution of number of mutational events for all SARS-CoV-2 genome samples analyzed. **(B)** Distributions of number of mutations for each sample, stratified per continent. The main boxplot rectangles are drawn between the 1st and 3rd quartile, with the median value indicated as a thick line. Boxplot whiskers fall on the closest point to the 1st/3rd quartile + 1.5 interquartile range as described in the R boxplot() function. The number in brackets after the continent name indicates the number of sequenced genomes. The horizontal red line indicates the average number of mutations per sample, worldwide. **(C)** As in **(B)**, with stratification performed country-wise, using the 40 countries with the highest number of sequenced genomes. The boxplot color indicates the country has a mutation rate higher (red) or lower (blue) than the world's average (Kolmogorov-Smirnov test *p* < 2.2 × 10^−16^ and absolute difference of averages between country and world higher than one).

Our analysis of 48,635 SARS-CoV-2 highlights a total of 353,341 mutation events compared to the NC_045512.2 Wuhan reference genome. Our results, event by event, are available as Supplementary File 5. While 256 samples, mostly originating from Asia, did not have any difference from the reference, 48,379 samples possessed at least one mutation. The number of mutations is relatively low, with mode per sample equaling 6, an average of 7.23, and very few samples having more than 15 events (Figure 1A). Overall, no continent differs significantly from the average mutation rate (Figure 1B), but there is a significant difference (one-way ANOVA *p* = 9.55 × 10^−205^) in the average number of mutations per sample between countries. Specifically, amongst the top 40 nations with the highest number of sequenced full viral genomes (Figure 1C), these countries have a slightly but significant higher number of observed mutations per sample, when compared to the world's average: India: (8.40), Congo (8.30), Bangladesh (9.83), and Kazakhstan (9.47). On the other hand, the sequences from the following countries show a significantly lower mutational burden: Germany (6.09), Japan (4.55), Italy (5.92), Greece (5.91), Hong Kong (5.00), and Kenya (5.38). One must bear in mind that some sampling biases may affect this comparison: for example, some countries have generated the highest number of sequences in the early phases of the pandemic, and may have therefore observed fewer mutations (for example, Italy has not shared any sequence in the months of May and June 2020, the last two considered in our analysis). On the other hand, one would expect China to have a lower number of mutations, being the likely point of origin of SARS-CoV-2 (Ceraolo and Giorgi, 2020), and indeed the distribution of mutations per sample seems to suggest that (Figure 1C); however, a small number of sequences carrying a very high number (>50) of mutations are associated to China, shifting the distribution for this country. Upon manual inspection, these sequences do not appear to share similarities between each other, and are likely the product of technical sequencing errors.

**Figure 2:**
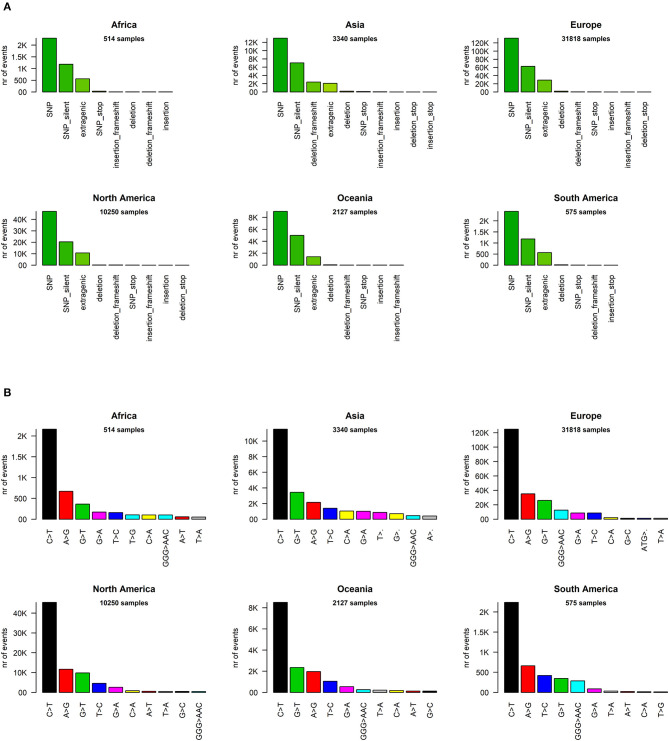
**(A)** Distribution of SARS-CoV-2 mutation classes in continents. “SNP,” “deletion,” and “insertion” terms without further specifications are intended as frameshift-preserving aa-changing events. **(B)** Continent-stratified distribution of SARS-CoV-2 mutation types. Colors are assigned randomly but preserved across panels to facilitate tracking of identical types across continents. Listed nucleotide changes represent those found in the positive-sense viral RNA. We indicate the thymine T letter for consistency with the NCBI reference sequence NC_045512.2, but the actual viral sequence will be factually represented by a U (uracil) as the RNA counterpart for thymine. Dots (‘·’) on the x-axis mutation type names indicate nucleotide deletion.

We analyzed the nature of each mutation, highlighting a massive prevalence of single-nucleotide polymorphisms (SNPs) over short insertion/deletion events (indels) worldwide (Supplementary File 6) and in every continent (Figure 2A). Worldwide, we observed 205,482 amino acid(aa)-changing SNP events (58.2% of the total), with fewer than half silent SNPs falling in coding regions (27.6%, with 97,573 events). There are 44,345 events in intergenic regions (12.6%), prevalently the 5′UTR and 3′UTR of the SARS-CoV-2 RNA sequence. Short frameshift deletions are the most common indel event in the SARS-CoV-2 population (0.8%), followed by in-frame deletions (3x deletions reducing the viral protein length without introducing stop codons), which account for 0.6% of all observed mutational events. SNPs generating a stop codon are also very rare (496 observed events, 0.1% of the total). Insertions are an extremely rare event, accounting for <0.1% of all SARS-CoV-2 mutations detected so far. Similar profiles and relative percentages are observed in all continents, suggesting a conserved molecular basis for SARS-CoV-2 evolution (Figure 2A).

We then classified the SARS-CoV-2 mutations according to their type, observing a prevalence of SNP transitions (purine->purine and pyrimidine->pyrimidine) over SNP transversions (purine->pyrimidine and vice versa), an observation that matches what was observed for SARS-CoV (Hu et al., 2003). The most common event, both worldwide and continent-wise, is by far the C>T transition, accounting for 55.1% of all observed worldwide viral mutations (Figure 2B, Supplementary File 6). The A>G transition is the second most common event worldwide (14.8%) and in Africa, Europe, and the Americas. The most common transversion, G>T, is the third most common event worldwide, with 42,408 occurrences (12.0%), but it is the second most common event in Asia and Oceania. The most common indel, the deletion of the ATG codon, is the 12th most common event worldwide, with a total of 1,298 occurrences, but it rises to the 9th most frequent in European genomes (Figure 2B). A peculiar multi-nucleotide event, the substitution of a GGG triplet with AAC, was also observed as the 5th most common event worldwide (4.0%, Supplementary File 6). As we will discuss later, this mutation type is mostly associated to a specific event affecting the Nucleocapsid locus, which characterizes the clade GR in the viral phylogenetic tree. It must be noted here that our choice of the “T” base notation, corresponding to thymine, was made for compatibility reasons with the NCBI NC_045512.2 reference genome notation, while the actual RNA base in the SARS-CoV-2 genome is a “U” (Uracil).

**Figure 3:**
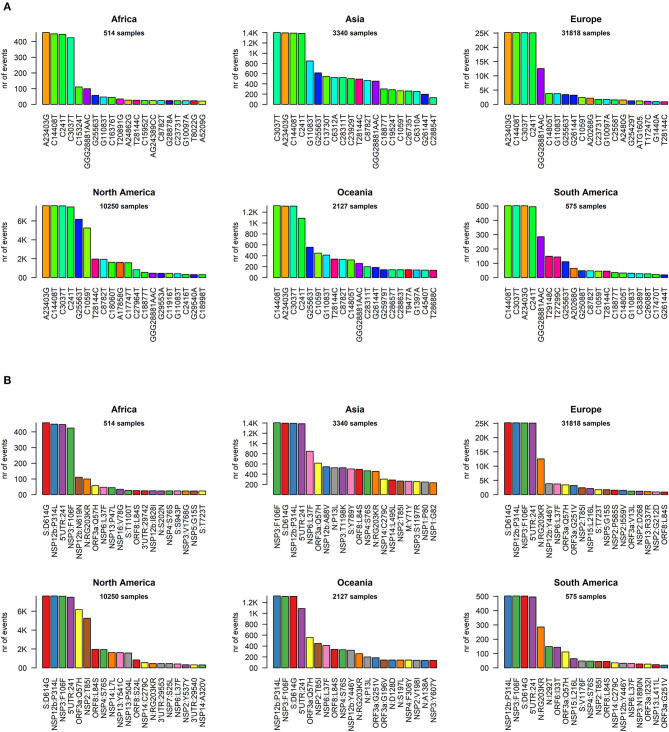
**(A)** Continent-stratified distribution of SARS-CoV-2 most frequent specific events, annotated as nucleotide coordinates over the reference genome NC_045512.2. Colors are assigned randomly but preserved across panels to facilitate tracking of identical types across continents. **(B)** Continent-stratified distribution of SARS-CoV-2 most frequent specific events, annotated protein changes using the format protein:mutation. Colors are assigned randomly but preserved across panels to facilitate tracking of identical types across continents.

We went into higher detail and analyzed the effects of each mutation on the protein sequences of SARS-CoV-2. Again, the profiles appear quite similar across continents. The most prevalent mutation in sequenced genomes worldwide is a transversion affecting the 23,403rd nucleotide adenosine (Supplementary File 6), transformed into a guanosine (A23403G), defining the so-called G-clade of SARS-CoV-2 genomes, prevalent in Europe (where overall the highest sequencing effort has been undertaken, and therefore the highest number of samples), Oceania, South America, and Africa (Figure 3A). This mutation causes a D614G (aspartate to glycine in protein position 614) aa-change of the Spike (S) protein, which is responsible for the initial entry of the virus in the cell via the ACE2 human receptor (Guzzi et al., 2020). However, this mutation is outside the observed Spike/ACE2 binding domain, roughly located between amino acids 330 and 530 (Wang et al., 2020). Three mutations show similar frequency with A23403G: C14408T, C241T, and C3037T (Figure 3A). As we will show later, these four mutations are almost always co-occurring in the same genomes, defining the major clade G observed in the viral population. In Asia, while the most common mutation was G11083T for samples sequenced between December 2019 and March 2020, recent sequencing efforts have highlighted a current profile similar to those of the other continents (Figure 3A).

The effect of the majority of SARS-CoV-2 nucleotide mutations is reflected in protein changes. We show, in Figure 3B, the most common mutations, in protein annotation, in the six continents, while in Table 1 we highlight the effect of the 20 most common mutations worldwide, in nucleotide and protein coordinates. The most common set of events is a quadruplet of mutations, corresponding to the G clade nucleotide mutations described before. Apart from the aforementioned D614G mutation observed in the Spike protein, the second most common amino acid changing mutation is P314L, affecting the Non-structural Protein 12 (NSP12), the viral RNA-dependent RNA polymerase. The other two mutations in the top four do not affect the protein sequence, as they are silent mutations targeting the 106th codon of NSP3 (a viral predicted phosphoesterase) and the 5′UTR in position 241.

**Table 1:** The 20 most frequent mutation events observed in sequenced SARS-CoV-2 genomes.

**Genomic coordinate**	**Effect on protein/UTR**	**Nr of samples**	**Class**	**Genomic region**
A23403G	S:D614G	36,500	aa-changing SNP	Spike protein
C14408T	NSP12b:P314L	36,444	aa-changing SNP	Non-structural protein 12, post-ribosomal frameshift (RNA-dependent RNA polymerase)
C3037T	NSP3:F106F	36,384	silent SNP	Non-structural protein 3 (predicted phosphoesterase)
C241T	5′UTR:241	36,007	5′UTR SNP	5′ UnTranslated Region
GGG28881AAC	N:RG203KR	14,095	aa-changing SNP triplet	Nucleocapsid protein
G25563T	ORF3a:Q57H	10,929	aa-changing SNP	ORF3a protein
C1059T	NSP2:T85I	8,451	aa-changing SNP	Non-structural protein 2
G11083T	NSP6:L37F	5,507	aa-changing SNP	Non-structural protein 6 (transmembrane protein)
C14805T	NSP12b:Y446Y	4,505	silent SNP	Non-structural protein 12, post-ribosomal frameshift (RNA-dependent RNA polymerase)
T28144C	ORF8:L84S	3,804	aa-changing SNP	ORF8 protein
G26144T	ORF3a:G251V	3,792	aa-changing SNP	ORF3a protein
C8782T	NSP4:S76S	3,743	silent SNP	Non-structural protein 4
A20268G	NSP15:L216L	2,479	silent SNP	Non-structural protein 15 (endoRNAse)
C18060T	NSP14:L7L	1,813	silent SNP	Non-structural protein 14 (3′-5′ exonuclease)
C23731T	S:T723T	1,799	silent SNP	Spike protein
G10097A	NSP5:G15S	1798	aa-changing SNP	Non-structural protein 5 (protease)
A17858G	NSP13:Y541C	1,780	aa-changing SNP	Non-structural protein 13
C17747T	NSP13:P504L	1,736	aa-changing SNP	Non-structural protein 13
C2558T	NSP2:P585S	1,701	aa-changing SNP	Non-structural protein 2
A2480G	NSP2:I559V	1,615	aa-changing SNP	Non-structural protein 2

*The acronym “aa” stands for “amino acid”*.

Other common mutations affecting protein sequence are N:RG203KR (in the Nucleocapsid protein N), induced by a tri-nucleotide mutation and determining a 2-amino acid change and mutations affecting the less characterized ORF3a, ORF8, NSP2, NSP6, and NSP13 proteins (Table 1). The G15S mutation in the viral protease NSP5 is the 16th most common event worldwide, with 1,798 samples affected (3.7%), however it seems to be too peripheric, in the protein sequence, to influence catalytic activity, and folding (Zhang et al., 2020).

**Figure 4:**
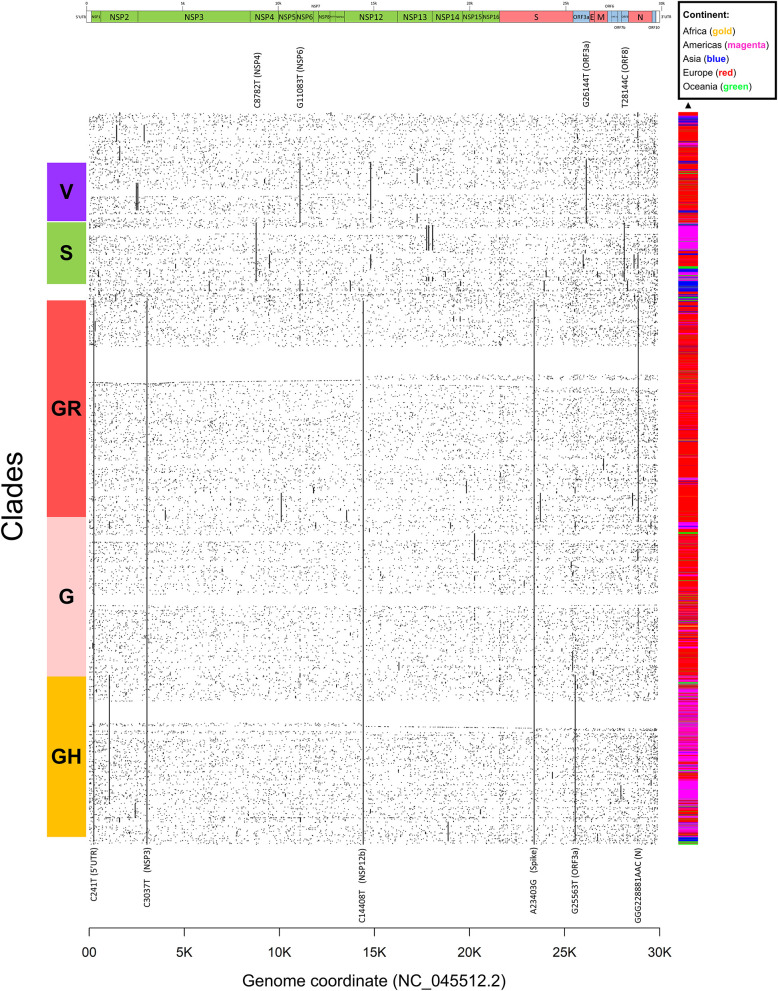
Dot mat showing as X-axis the 29,903 nucleotide positions (sorted from left, 5′ to right, 3′) of SARS-CoV-2, and as Y axis the 48,635 genomes analyzed in this study. The genomic sequences were clustered using simple correlation followed by the “complete” clustering algorithm. Coding sequence regions are shown at the top. To the right of the plot, we assigned a color to each sample according to the continent of origin. On the left, we manually annotated the groups according to the known GISAID clades (G, GH, GR, S, and V) and the mutations that named them. Labels of clade-defining mutations are placed on the corresponding genomic coordinate.

We proceeded then to analyze the distribution of mutation groups rather than individual events, in order to observe their phylogenetic groups and geographical and temporal distributions. Our observation on co-occurring mutations (Figure 4) matches the current phylogenetic classification defined by the GISAID consortium (Table 2). Specifically, the four mutations C241T, C3037T, C14408T, and A23403G are observed in all samples from the clade “G” (named after the Spike D614G mutation) and its two derivative GH (further characterized by the ORF3a:Q57H mutation) and GR (affected by the trinucleotide mutation in the Nucleocapsid gene, inducing a RG203KR mutation).

**Table 2:** Current definition of characterizing mutations of SARS-CoV-2 phylogenetic categorization systems (GISAID clades and PANGOLIN lineages).

**GISAID clade**	**PANGOLIN lineage**	**Nucleotide features**	**Corresponding effects on protein sequence**
G	B.1	C241T C3037T C14408T A23403G	5′UTR NSP3:F106F NSP12b:P314L S:D614G
GH	B.1.*	C241T C3037T C14408T A23403G G25563T	5′UTR NSP3:F106F NSP12b:P314L S:D614G ORF3a:Q57H
GR	B.1.1	C241T C3037T C14408T A23403G GGG28881AAC	5′UTR NSP3:F106F NSP12b:P314L S:D614G N:RG203KR
S	A	C8782T T28144C	NSP4:S76S ORF8:L84S
V	B.2	G11083T G26144T	NSP6:L37F ORF3a:G251V
L		Reference in all nts defining clades G, GH, GR, S, and V	
O		Others	

Other two major clades are called “S,” named after the mutation in ORF8 L84S (Ceraolo and Giorgi, 2020), also characterized by a silent C8782T genomic mutation, and “V,” from the ORF3a:G251V mutation, almost always co-occurring with the NSP6:L37F event, and identified by early phylogenetic studies (Forster et al., 2020). The original lineage “L,” corresponding to the reference genome NC_045512.2, is populated in our study by all genomes carrying reference alleles for all loci defined in clades G, GH, GR, S, and V (Table 2). Finally, a general group for other sequences not matching any of these criteria (e.g., other alleles or combinations) is defined here as “O” clade. Clustering all genomes clearly highlights the five major phylogenetic groups G, GH, GR, S, and V and their characterizing mutations (Figure 4), as well as more nascent clades (e.g., in the GH clade, further split by a novel mutation in the NSP2 locus, C1059T), and a general distribution of non-recurring mutations for the majority of sequences. There are, however, a few hundreds of highly “clean” sequences (e.g., for clade GR), characterized by the exclusive presence of the clade-characterizing mutations.

Generally, the G and GR clades are prevalently present in Europe, while the clade S and GH have been mostly observed in the Americas (Figure 4). The “L” reference clade is mostly represented by sequences from Asia, where the virus likely originated (Andersen et al., 2020). In Table 2, we also report, for reference and completeness, the corresponding nomenclature used by the PANGOLIN phylogenetic classification (Rambaut et al., 2020).

**Figure 5:**
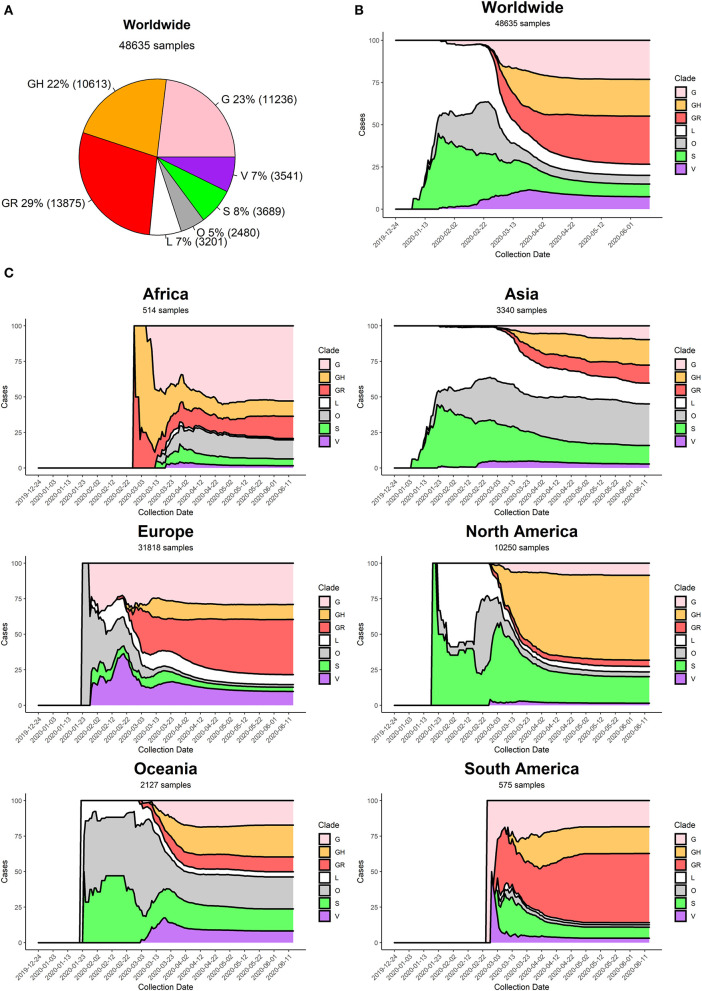
**(A)** Distribution of SARS-CoV-2 clades in the World at the time of writing (26 June 2020). **(B)** Stacked area chart of relative SARS-CoV-2 clade frequency (y-axis) over time (x-axis) worldwide. **(C)** Stacked area charts of relative SARS-CoV-2 clade frequency over time in six continents.

Currently, the G clade and its offspring, GH and GR, are the most common clades amongst the sequenced SARS-CoV-2 genomes, globally accounting for 74% of all world sequences (Figure 5A). Specifically, the GR clade, carrying the combination of Spike D614G and Nucleocapsid RG203KR mutations, is currently the most common representative of the SARS-CoV-2 population worldwide. The original viral strain, represented by clade L, still accounts for 7% of the sequenced genomes, and the other derived clades S and V have similar frequencies in the global dataset.

At the beginning of the COVID-19 pandemic (December 2019) the most commonly retrieved genome was the reference one (clade L), but the first mutated virus appeared in sequence databases at the beginning of 2020 (clade S) alongside other, less clearly defined, sequences (generic clade O). The clade V (mutated in NSP6 and ORF3a) appeared around mid-January 2020, around the same time as the original clade G (Figure 5B). The first detection of subclades GH and GR can be placed more than a month later, at the end of February 2020. Sequencing efforts, mostly located in North America and Europe, have demonstrated an ever-increasing frequency of G, GH, and GR genomes, which have gradually become the most represented sequences in the GISAID database (Figure 5B).

Our analysis highlights pivotal differences in clade distribution over time between continents (Supplementary File 7, Figure 5C). Currently, the vastly prevalent genome in North America is GH (mutations in Spike D614G and ORF3a Q57H), accounting for more than 50% sequences submitted. In Europe and South America, the most frequent clades are GR, while in Oceania there seems to be the most balanced co-existence of all observed clades. Africa shows a prevalence of clade G. It is interesting to note that Asia, initially characterized by reference sequences, is currently observing a rise in G, GH, and GR genomes, which gained ground in the continent at the beginning of March 2020, more than 1 month after the appearance of these clades in Europe (Figure 5C).

We provide, as Supplementary File 8, also a country-wise analysis of the 32 countries with most SARS-CoV-2 full genome sequences available. As a general observation, countries tend to follow the general trend of their continent, with a few notable exceptions. China, for example, has produced almost no sequences belonging to clades G and derivatives. Moreover, some European countries have a prevalence of GH genomes (Denmark, France), while others show higher numbers of GR (United Kingdom, Portugal). The currently predominant clade in the United States of America is GH, like Israel and Saudi Arabia, while the most common genomes in Russia and Brazil belong to clade GR.

Generally speaking, we observe an increase over time in G clade genomes, and its derivatives GH and GR, paired by a gradual disappearance of clades L and V. Clade S, while declining, seems to be still accounting for a significant minority of sequenced genomes, especially in the United States of America and Spain.

**Figure 6:**
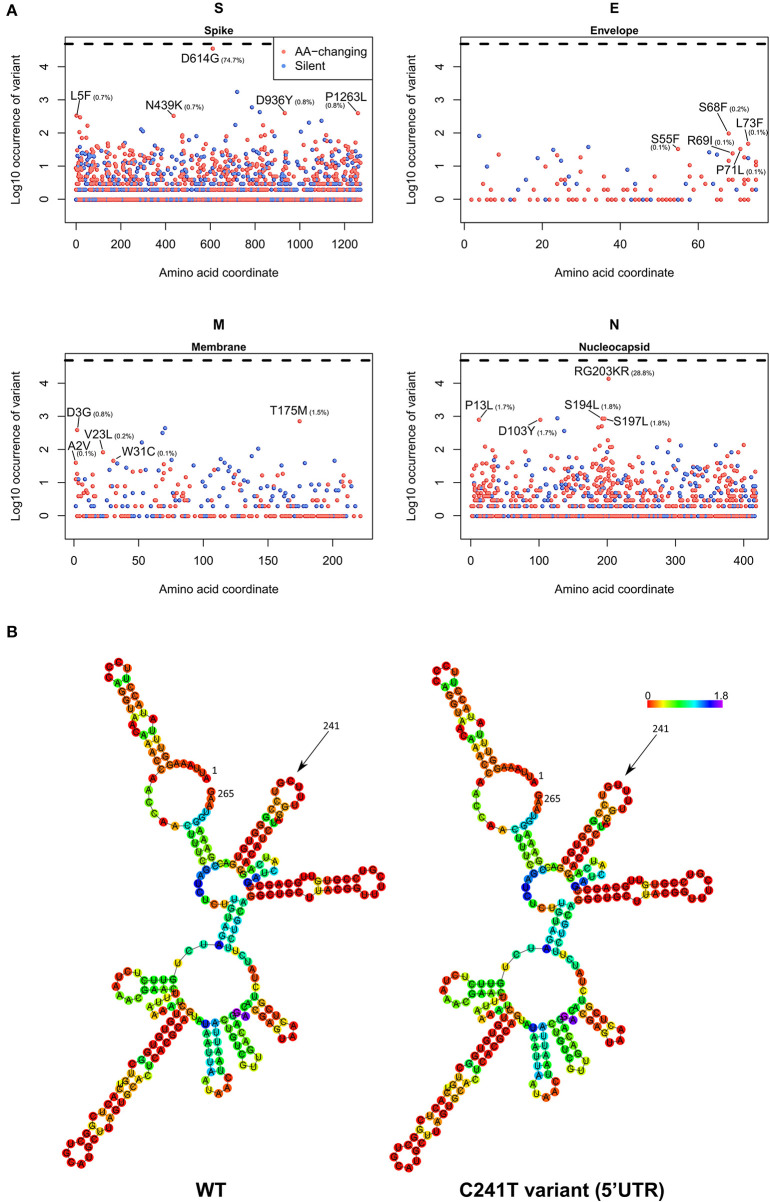
**(A)** Occurrence of mutations in the four SARS-CoV-2 structural proteins S (Spike), E (Envelope), M (Membrane), and N (Nucleocapsid). On the x-axis, the amino acid coordinate of the mutation. On the y-axis, the Log10 of the number of samples where the mutations have been observed, worldwide. The horizontal dashed line indicates the maximum (Log10 of all the 48,635 samples). In blue, silent mutations, and in red, mutations affecting the protein sequence. The frequency (in percentage) of the top 5 aa-changing mutations is also indicated. **(B)** Dot-bracket notation of minimum free energy prediction of the secondary structure of SARS-CoV-2 5′UTR (nt 1-265), WT (left) and C241T variant (right). Base reliability is expressed as positional entropy and colored accordingly.

As a final part of our analysis, we analyzed the effects of mutations in the 26 SARS-CoV-2 proteins, producing a map of all the most frequent observed aa-changing mutations (Supplementary File 9). All proteins are affected by at least one recurring (>75 observations), even if rarer, non-silent mutation. In general, mutations seem to be distributed uniformly across the viral genome, with the obvious exception of highly frequent clade-defining mutations. We analyzed in detail the four structural proteins S (Spike), E (Envelope), M (Membrane), and N (Nucleocapsid) in Figure 6A. The Spike protein, apart from the discussed D614G mutation, has no other event present in more than 1% of the viral population; amongst the top 5, a N439K variant located in the Spike/ACE2 interaction domain is observed in 0.7% of the viruses. The Envelope protein appears to be the most conserved, with the most frequent mutations present in the C-terminus and never present in more than 0.2% of the population. More than 1% of sequenced viruses show a T175M mutation in the Membrane protein. The Nucleocapsid protein, apart from the clade GR-defining RG203KR mutation, has several non-silent mutations above the threshold of 1% frequency in the population, specifically P13L, D103Y, S194L, and S197L (Figure 6A).

We also analyzed the C241T mutation, located in the SARS-CoV-2 5′UTR. While not inducing a change in protein sequence, we postulated that this event may have effects in the secondary RNA structure, therefore influencing the rate of RNA replication and therefore the speed of the viral infection cycle (Kim et al., 2020). Our prediction, based on the Vienna RNA suite (Figure 6B) shows no significant difference in the secondary structure of the wild-type (WT) genome and the C241T variant, since this nucleotide is not participating in any hydrogen bond with other nucleotides.

## Discussion

Our analysis, based on 48,635 samples, confirms a low mutation rate of the virus, with an average of 7.23 mutations per sample with respect to the reference SARS-CoV-2 genome sequences. One *caveat* of our estimate is that it is based on assembled genomes, not on raw Illumina, Oxford Nanopore, or Sanger sequencing data. This made it impossible to analyze e.g., the presence of viral subpopulations within the same patient and to evaluate the complex evolutionary events within the SARS-CoV-2 quasispecies (Knyazev et al., 2020). It is therefore likely that the actual mutation rate of SARS-CoV-2 is higher than 7.23, which is calculated from reported sequences of the sole dominant population. This is further sustained by the recent evidence of intense RNA editing in the SARS-CoV-2 genome, fueled by the human host cell APOBEC mechanism (Milewska et al., 2018; Di Giorgio et al., 2020), which can also explain the prevalence of transitions as the prevalently observed mutational events.

While few, the existing detected mutations allow to group the samples into five distinct clades, G, GH, GR, S, and V, characterized by a collection of specific mutations. The clades can be further characterized by most recent mutations and will likely be split even further in the future.

The aa-changing SNPs are the most prevalent mutational events, followed by silent SNPs and extragenic (mostly 5′UTR) SNPs. The silent events may not determine an immediate effect on the protein sequences, but they have repercussions as they may change the codon usage and translation efficiency. In the case of the 5′UTR SNPs, mutations may affect the transcription and replication rates of the virus, or the folding of the genomic ssRNA, processes that have been only recently and only partially elucidated (Kim et al., 2020).

The early studies currently performed on SARS-CoV-2 transcriptome dynamics may also suggest mechanisms for mutation onset, which our study shows being prevalently single-nucleotide transitions. This phenomenon can be associated to defective efficiency of the viral RNA-depedent RNA polymerase or, as recently suggested, by mechanisms of RNA editing triggered by the host cell as a defense mechanism (Di Giorgio et al., 2020). Whatever the origin, SARS-CoV-2 tends to retain its genomic integrity across propagation, with almost no reported large indels across sequenced genomes (the largest reported being a unique 80-nucleotide deletion in ORF7a, in Arizona sample EPI_ISL_424669 – Supplementary File 5).

Further studies combining genomic details with epidemiological information and clinical features of COVID-19 patients may be extremely useful to identify strategies and therapies that can help to reduce the burden of this disease. There is currently little evidence on the clinical and molecular differences between the circulating clades of SARS-CoV-2; for example, one study has shown that the D614G mutation in the Spike protein may be associated to higher case fatality rates (Becerra-Flores and Cardozo, 2020). However, as this coronavirus continues to evolve, surely new features will emerge or mutate alongside the genomic sequences, with clinical and pharmacological repercussions.

The emergence of new mutations may force the development of new antiviral therapies, as well as the adaptation of current ones to tackle the new molecular structures of the virus. For example, the development of protein-based and RNA-based vaccines based on the SARS-CoV-2 Spike region (Amanat and Krammer, 2020) will have to take into account the observed diversity of the Spike protein. The prevalent Spike D614G mutation does not seem to affect the interaction domain with ACE2 (Wang et al., 2020), responsible for the viral entry into epithelial cells (Guzzi et al., 2020), but other mutations are currently located in that domain, such as N439K, present in 0.7% of the sequenced SARS-CoV-2 genomes. Our analysis in Figure 4 shows that new mutations and clades are emerging beyond the current clade categorization and will likely expand if they confer an evolutionary advantage to SARS-CoV-2.

Constant monitoring of mutations will also be pivotal in tracking the movement of the virus between individuals and across geographical areas. For example, our descriptive analysis of clade prevalence over time (Figure 5) shows the birth of the original L clade in Asia (China) in December 2019, followed by the appearance of the G clade in Europe in January 2020. G and G-derived clades have then reached North America and Asia in March 2020 and are currently the fastest growing viral subpopulation worldwide. Tracking viral evolution must benefit however from constant monitoring of the SARS-CoV-2 genomic sequences, with *ad-hoc* epidemiological and genomic online resources that go beyond the scope of this publication (Hufsky et al., 2020; Mercatelli et al., 2020). One of such tools is NextStrain (Hadfield et al., 2018), which also allows for scalable phylogenetic analyses and real time tracking of specific mutations.

## Data Availability Statement

Publicly available datasets were analyzed in this study. This data can be found here: https://www.gisaid.org/.

## Author Contributions

FG designed the study. FG and DM performed research, analyzed data, and wrote the manuscript. All authors contributed to the article and approved the submitted version.

## Conflict of Interest

The authors declare that the research was conducted in the absence of any commercial or financial relationships that could be construed as a potential conflict of interest.

## Supplementary Material

Supplementary File 1 GISAID acknowledgment table reporting the geographic origin and contributions of genomes analyzed in this study.

Supplementary File 2 Annotation of samples used in this dataset.

Supplementary File 3 Annotation of NC_045512.2 SARS-CoV-2 Wuhan genome sequence (GFF3 format).

Supplementary File 4 Bash/R scripts used to generate and annotate genome variants.

Supplementary File 5 Full annotation of all mutations identified by this study. Columns are described here. Sample: GISAID sample id; refpos: position in the NC_045512.2 reference genome; refvar: nucleotide composition of the reference at refpos coordinate (a “.” indicates an insertion); qvar: variant in the query sample (a “.” indicates a deletion); qlength: length of the query genome (reference genome is always 29,903 nucleotides long); region: region annotated in the event position (coding sequence, intergenic or UTR); variant: either a protein change (shown as aminoacid code) or the genomic position (if the event affects a non-coding region); varclass: variant class (as in Figure 2A); annotation: full name of the protein coded by the affected region (if coding); varname: full name of protein variant; varclade: full name of nucleotide variant.

Supplementary File 6 Worldwide analysis of most frequent mutations categorized per class, type, nucleotide, and protein events.

Supplementary File 7 Distribution of SARS-CoV-2 clades in all continents at the time of writing.

Supplementary File 8 Stacked area charts of relative SARS-CoV-2 clade frequency (y-axis) over time (x-axis) in the 32 countries with the highest number of full genome sequences.

Supplementary File 9 Table of aa-changing mutations, categorized by protein and sorted by number of samples where the mutation has been observed.

## Abbreviations

AA: amino acid
COVID-19: Coronavirus Disease 2019
GISAID: Global Initiative on Sharing All Influenza Data
Indel: insertion/deletion event
NSP: non-structural protein
ORF: open reading frame
S: SARS-CoV-2 spike protein
SARS-CoV-2: Severe Acute Respiratory Syndrome, Coronavirus 2
SNP: single nucleotide polymorphism.
