# Supplementary material for: Geographic and Genomic Distribution of SARS-CoV-2 Mutations
Source: Front Microbiol. 2020 Jul 22;11:1800. doi: 10.3389/fmicb.2020.01800 (PMC7387429; doi:10.3389/fmicb.2020.01800)
Supplement: Supplementary File 3 — Annotation of NC_045512.2 SARS-CoV-2 Wuhan genome sequence (GFF3 format). [file Table_3.DOCX]

**Supplementary File**

**Annotation of SARS-CoV-2 NC_045512.2 genome (GFF3 format)**

##sequence-region NC_045512.2 1 29903

##species https://www.ncbi.nlm.nih.gov/Taxonomy/Browser/wwwtax.cgi?id=2697049

NC_045512.2 Giorgi CDS 266 805 . + . NSP1 Leader protein

NC_045512.2 Giorgi CDS 806 2719 . + . NSP2 Non-Structural protein 2

NC_045512.2 Giorgi CDS 2720 8554 . + . NSP3 Predicted phosphoesterase, papain-like proteinase

NC_045512.2 Giorgi CDS 8555 10054 . + . NSP4 Transmembrane protein

NC_045512.2 Giorgi CDS 10055 10972 . + . NSP5 3C-like proteinase

NC_045512.2 Giorgi CDS 10973 11842 . + . NSP6 Transmembrane protein

NC_045512.2 Giorgi CDS 11843 12091 . + . NSP7 Non-Structural protein 7

NC_045512.2 Giorgi CDS 12092 12685 . + . NSP8 Non-Structural protein 8

NC_045512.2 Giorgi CDS 12686 13024 . + . NSP9 ssRNA-binding protein

NC_045512.2 Giorgi CDS 13025 13441 . + . NSP10 Growth-factor-like protein

NC_045512.2 Giorgi CDS 13442 13468 . + . NSP12a RNA-dependent RNA polymerase, pre-ribosomal frameshift

NC_045512.2 Giorgi CDS 13468 16236 . + . NSP12b RNA-dependent RNA polymerase, post-ribosomal frameshift

NC_045512.2 Giorgi CDS 16237 18039 . + . NSP13 Helicase

NC_045512.2 Giorgi CDS 18040 19620 . + . NSP14 3'-to-5' exonuclease

NC_045512.2 Giorgi CDS 19621 20658 . + . NSP15 endoRNAse

NC_045512.2 Giorgi CDS 20659 21552 . + . NSP16 2'-O-ribose methyltransferase

NC_045512.2 Giorgi CDS 21563 25384 . + . S Spike

NC_045512.2 Giorgi CDS 25393 26220 . + . ORF3a ORF3a protein

NC_045512.2 Giorgi CDS 26245 26472 . + . E Envelope

NC_045512.2 Giorgi CDS 26523 27191 . + . M Membrane

NC_045512.2 Giorgi CDS 27202 27387 . + . ORF6 ORF6 protein

NC_045512.2 Giorgi CDS 27394 27759 . + . ORF7a ORF7a protein

NC_045512.2 Giorgi CDS 27756 27887 . + . ORF7b ORF7b protein

NC_045512.2 Giorgi CDS 27894 28259 . + . ORF8 ORF8 protein

NC_045512.2 Giorgi CDS 28274 29533 . + . N Nucleocapsid protein

NC_045512.2 Giorgi CDS 29558 29674 . + . ORF10 ORF10 protein
