# Supplementary material for: Geographic and Genomic Distribution of SARS-CoV-2 Mutations
Source: Front Microbiol. 2020 Jul 22;11:1800. doi: 10.3389/fmicb.2020.01800 (PMC7387429; doi:10.3389/fmicb.2020.01800)
Supplement: Supplementary File 4 — Bash/R scripts used to generate and annotate genome variants. [file Table_4.DOCX]

**Supplementary File**

***Pipeline for the annotation of SARS-CoV-2 genome sequences***

##########################################

### COVID-19 genome mutation annotator ###

##########################################

## By Federico M. Giorgi

#################################

###### BASH section

### Download SARS-CoV-2 sequences in FASTA format

input=input.fasta

### Run nucmer to obtain variant file

ref=NC_045512.2.fa # The reference SARS-CoV-2 Wuhan Genome

dos2unix $input

nucmer --forward -p nucmer $ref $input

show-coords -r -c -l nucmer.delta > nucmer.coords

show-snps nucmer.delta -T -l > nucmer.snps

#################################

###### R section

# Load variant list

nucmer<-read.delim("nucmer.snps",as.is=TRUE,skip=4,header=FALSE)

colnames(nucmer)<-c("rpos","rvar","qvar","qpos","","","","","rlength","qlength","","","rname","qname")

rownames(nucmer)<-paste0("var",1:nrow(nucmer))

nrow(nucmer) # 69989

# Fix IUPAC codes

table(nucmer$qvar)

nucmer<-nucmer[!nucmer$qvar%in%c("B","D","H","K","M","N","R","S","V","W","Y"),]

nrow(nucmer) # 69980

### Aminoacid variant list ----

# Load reference sequence

library(seqinr)

library(Biostrings)

refseq<-read.fasta("NC_045512.2.fa",forceDNAtolower=FALSE)[[1]]

# Load GFF3

gff3<-read.delim("NC_045512.2_annot.gff3",as.is=TRUE,skip=2,header=FALSE)

annot<-setNames(gff3[,10],gff3[,9])

### Merge neighboring events ----

samples<-unique(nucmer$qname)

length(samples) # 9884

pb<-txtProgressBar(0,length(samples),style=3)

for (pbi in 1:length(samples)){ # This will update the nucmer object

sample<-samples[pbi]

allvars<-nucmer[nucmer$qname==sample,]

snps<-allvars[(allvars[,"rvar"]!=".")&(allvars[,"qvar"]!="."),]

inss<-allvars[(allvars[,"rvar"]=="."),]

dels<-allvars[(allvars[,"qvar"]=="."),]

# Merge insertions

prevqpos<-0

prevrowname<-NULL

remove<-c()

i<-1

corrector<-0

while(i<=nrow(inss)){

rpos<-inss[i,"rpos"]

rvar<-inss[i,"rvar"]

qvar<-inss[i,"qvar"]

qpos<-inss[i,"qpos"]

if((qpos!=1)&(qpos==(prevqpos+1+corrector))){

inss<-inss[-i,]

inss[prevrowname,"qvar"]<-paste0(inss[prevrowname,"qvar"],qvar)

corrector<-corrector+1

i<-i-1

} else {

corrector<-0

prevrowname<-rownames(inss)[i]

prevqpos<-qpos

}

i<-i+1

}

# Merge deletions

prevqpos<-0

prevrowname<-NULL

remove<-c()

i<-1

while(i<=nrow(dels)){

rpos<-dels[i,"rpos"]

rvar<-dels[i,"rvar"]

qvar<-dels[i,"qvar"]

qpos<-dels[i,"qpos"]

if((qpos!=1)&(qpos==(prevqpos))){

dels<-dels[-i,]

dels[prevrowname,"rvar"]<-paste0(dels[prevrowname,"rvar"],rvar)

i<-i-1

} else {

prevrowname<-rownames(dels)[i]

prevqpos<-qpos

}

i<-i+1

}

# Merge SNPs

prevqpos<-0

prevrowname<-NULL

remove<-c()

i<-1

corrector<-0

while(i<=nrow(snps)){

rpos<-snps[i,"rpos"]

rvar<-snps[i,"rvar"]

qvar<-snps[i,"qvar"]

qpos<-snps[i,"qpos"]

if((qpos!=1)&(qpos==(prevqpos+1+corrector))){

snps<-snps[-i,]

snps[prevrowname,"rvar"]<-paste0(snps[prevrowname,"rvar"],rvar)

snps[prevrowname,"qvar"]<-paste0(snps[prevrowname,"qvar"],qvar)

corrector<-corrector+1

i<-i-1

} else {

corrector<-0

prevrowname<-rownames(snps)[i]

prevqpos<-qpos

}

i<-i+1

}

# Remerge back

allvars2<-rbind(snps,inss,dels)

remove<-setdiff(rownames(allvars),rownames(allvars2))

nucmer<-nucmer[setdiff(rownames(nucmer),remove),]

nucmer[rownames(allvars2),]<-allvars2

setTxtProgressBar(pb,pbi)

}

### Provide effect of each SNP and indel ----

header<-c("sample","refpos","refvar","qvar","qpos","qlength","protein","variant","varclass","annotation")

results<-matrix(NA,ncol=length(header),nrow=0)

colnames(results)<-header

samples<-unique(nucmer$qname)

pb<-txtProgressBar(0,length(samples),style=3)

for (pbi in 1:length(samples)){ # This will update the nucmer object

sample<-samples[pbi]

allvars<-nucmer[nucmer$qname==sample,]

# Check changes in query protein sequence according to variants

for(i in 1:nrow(allvars)){ # Assuming they are sorted numerically

nucline<-allvars[i,]

rpos<-nucline[1,"rpos"]

rvar<-nucline[1,"rvar"]

qvar<-nucline[1,"qvar"]

qpos<-nucline[1,"qpos"]

qlength<-nucline[1,"qlength"]

# Match over GFF3 annotation

a<-rpos-gff3[,4]

b<-rpos-gff3[,5]

signs<-sign(a)*sign(b)

w<-which(signs==-1)

# Outside genes scenarios

if(length(w)==0){

if(rpos<gff3[1,4]){

protein<-"5'UTR";output<-c(rpos,"extragenic")

} else if(rpos>gff3[1,5]){

protein<-"3'UTR";output<-c(rpos,"extragenic")

} else {

protein<-"intergenic";output<-c(rpos,"extragenic")

}

} else{ # Inside genes scenario

start<-gff3[w,4]

end<-gff3[w,5]

protein<-gff3[w,9]

refdnaseq<-DNAString(paste0(refseq[start:end],collapse=""))

refpepseq<-Biostrings::translate(refdnaseq)

refpepseq<-strsplit(as.character(refpepseq),"")[[1]]

if(qvar=="."){ # Deletion scenario

if((nchar(rvar)%%3)!=0){ # Deletion frameshift scenario

mutpos<-round((rpos-start+1)/3)

output<-c(paste0(refpepseq[mutpos],mutpos),"deletion_frameshift")

} else { # In-frame deletion

varseq<-refseq

varseq<-varseq[-(rpos:(rpos+nchar(rvar)-1))]

varseq<-varseq[start:(end-nchar(rvar))]

vardnaseq<-DNAString(paste0(varseq,collapse=""))

varpepseq<-Biostrings::translate(vardnaseq)

varpepseq<-strsplit(as.character(varpepseq),"")[[1]]

for(j in 1:length(refpepseq)){

refj<-refpepseq[j]

varj<-varpepseq[j]

if(refj!=varj){

if(varj=="*"){

output<-c(paste0(refj,j),"deletion_stop")

} else {

output<-c(paste0(refj,j),"deletion")

}

break()

}

}

}

} else if(rvar=="."){ # Insertion scenario

if((nchar(qvar)%%3)!=0){ # Insertion frameshift scenario

mutpos<-round((rpos-start+1)/3)

output<-c(paste0(refpepseq[mutpos],mutpos),"insertion_frameshift")

} else { # In-frame insertion

varseq<-c(refseq[1:rpos],strsplit(qvar,"")[[1]],refseq[(rpos+1):length(refseq)])

varseq<-varseq[start:(end+nchar(qvar))]

vardnaseq<-DNAString(paste0(varseq,collapse=""))

varpepseq<-Biostrings::translate(vardnaseq)

varpepseq<-strsplit(as.character(varpepseq),"")[[1]]

for(j in 1:length(refpepseq)){

refj<-refpepseq[j]

varj<-varpepseq[j]

if(refj!=varj){

nr_aa_inserted<-nchar(qvar)/3

multivarj<-varpepseq[j:(j+nr_aa_inserted-1)]

if(any(multivarj=="*")){

multivarj<-paste0(multivarj,collapse="")

output<-c(paste0(multivarj,j),"insertion_stop")

} else{

multivarj<-paste0(multivarj,collapse="")

output<-c(paste0(multivarj,j),"insertion")

}

break()

}

}

}

} else { # SNP scenario

if(nchar(qvar)==1){ # Single nucleotide scenario

varseq<-refseq

varseq[rpos]<-qvar

varseq<-varseq[start:end]

vardnaseq<-DNAString(paste0(varseq,collapse=""))

varpepseq<-Biostrings::translate(vardnaseq)

varpepseq<-strsplit(as.character(varpepseq),"")[[1]]

mutpos<-which(varpepseq!=refpepseq)

if(length(mutpos)==0){ # Silent SNP scenario

mutpos<-round((rpos-start+1)/3)

refaa<-refpepseq[mutpos]

varaa<-varpepseq[mutpos]

output<-c(paste0(refaa,mutpos,varaa),"SNP_silent")

} else { # Changed aa scenario

refaa<-refpepseq[mutpos]

varaa<-varpepseq[mutpos]

if(varaa=="*"){

output<-c(paste0(refaa,mutpos,varaa),"SNP_stop")

} else {

output<-c(paste0(refaa,mutpos,varaa),"SNP")

}

}

} else { # Multiple neighboring nucleotides

varlength<-nchar(qvar)

varseq<-refseq

varseq[rpos:(rpos+varlength-1)]<-strsplit(qvar,"")[[1]]

varseq<-varseq[start:end]

vardnaseq<-DNAString(paste0(varseq,collapse=""))

varpepseq<-Biostrings::translate(vardnaseq)

varpepseq<-strsplit(as.character(varpepseq),"")[[1]]

mutpos<-which(varpepseq!=refpepseq)

if(length(mutpos)==0){ # Silent SNP scenario

mutpos<-round((rpos-start+1)/3)

refaa<-refpepseq[mutpos]

varaa<-varpepseq[mutpos]

output<-c(paste0(refaa,mutpos,varaa),"SNP_silent")

} else { # Changed aa scenario

refaa<-paste0(refpepseq[mutpos],collapse="")

varaa<-paste0(varpepseq[mutpos],collapse="")

if(any(varaa=="*")){

output<-c(paste0(refaa,mutpos[1],varaa),"SNP_stop")

} else {

output<-c(paste0(refaa,mutpos[1],varaa),"SNP")

}

}

}

}

}

results<-rbind(results,c(sample,rpos,rvar,qvar,qpos,qlength,protein,output,annot[protein]))

}

setTxtProgressBar(pb,pbi)

}

results<-as.data.frame(results,stringsAsFactors=FALSE)

write.csv(results,file="covid_annot.csv",row.names = FALSE)

save(results,file="covid_annot.rda")

### Describe Results ----

png("covid_annot.png",w=3000,h=2000,res=300)

par(mfrow=c(2,3))

# Most mutated samples

occ<-sort(table(results$sample),dec=TRUE)[1:20]

par(las=2,mar=c(15,5,5,1))

barplot(occ,ylab="nr of mutations",main="Most mutated samples",col=heat.colors(length(occ)))

# Mutations per sample

occ<-table(table(results$sample))

par(las=2,mar=c(5,5,5,1))

barplot(occ,xlab="nr of mutations",main="Overall mutations per sample",col="cornflowerblue")

# Variant classes

occ<-sort(table(results$varclass),dec=TRUE)

par(las=2,mar=c(8,5,5,1))

barplot(occ,ylab="nr of events",main="Most frequent events per class",col=heat.colors(length(occ)))

# Variant class (A/T, etc)

occ<-sort(table(apply(results[,c("refvar","qvar")],1,paste0,collapse=">")),dec=TRUE)[1:20]

par(las=2,mar=c(5,5,5,1))

barplot(occ,ylab="nr of samples",main="Most frequent per type",col=heat.colors(length(occ)))

# Nucleotide events

occ<-sort(table(apply(results[,c("refvar","refpos","qvar")],1,paste0,collapse="")),dec=TRUE)[1:10]

par(las=2,mar=c(8,5,5,1))

barplot(occ,ylab="nr of samples",main="Most frequent events (nucleotide)",col=heat.colors(length(occ)))

# Protein events

occ<-sort(table(apply(results[,c("protein","variant")],1,paste0,collapse=":")),dec=TRUE)[1:10]

par(las=2,mar=c(8,5,5,1))

barplot(occ,ylab="nr of samples",main="Most frequent events (protein)",col=terrain.colors(length(occ)))

dev.off()
