# Supplementary material for: Geographic and Genomic Distribution of SARS-CoV-2 Mutations
Source: Front Microbiol. 2020 Jul 22;11:1800. doi: 10.3389/fmicb.2020.01800 (PMC7387429; doi:10.3389/fmicb.2020.01800)

Worldwide Analysis of most frequent mutations, sorted by number of events.

Total mutations: 353,341

| **Mutation classes** | |
| --- | --- |
| **Class** | **nr. events** |
| SNP | 205482 |
| SNP_silent | 97573 |
| extragenic | 44345 |
| deletion_frameshift | 2887 |
| deletion | 2280 |
| SNP_stop | 496 |
| insertion_frameshift | 153 |
| insertion | 113 |
| deletion_stop | 10 |
| insertion_stop | 2 |
|  |  |
|  |  |
| **Most frequent mutation types** |  |
| **Type** | **nr. events** |
| C>T | 194775 |
| A>G | 52326 |
| G>T | 42408 |
| T>C | 16260 |
| GGG>AAC | 14095 |
| G>A | 13047 |
| C>A | 4382 |
| T>A | 2204 |
| G>C | 2052 |
| A>T | 2032 |
| A>C | 1421 |
| ATG>. | 1298 |
| T>G | 1221 |
| T>. | 941 |
| G>. | 799 |
| A>. | 498 |
| C>. | 478 |
| C>G | 391 |
| AAGTCATTT>. | 176 |
| TG>AA | 112 |

| **Most frequent specific events (nucleotide)** | |
| --- | --- |
| **Event** | **nr. occurrences** |
| A23403G | 36500 |
| C14408T | 36436 |
| C3037T | 36384 |
| C241T | 35994 |
| GGG28881AAC | 14095 |
| G25563T | 10926 |
| C1059T | 8449 |
| G11083T | 5507 |
| C14805T | 4505 |
| T28144C | 3804 |
| G26144T | 3792 |
| C8782T | 3743 |
| A20268G | 2479 |
| C18060T | 1813 |
| C23731T | 1799 |
| G10097A | 1798 |
| A17858G | 1780 |
| C17747T | 1736 |
| C2558T | 1701 |
| A2480G | 1615 |

| **Most frequent specific events (protein)** | |
| --- | --- |
| **Event** | **nr. occurrences** |
| S:D614G | 36500 |
| NSP12b:P314L | 36444 |
| NSP3:F106F | 36384 |
| 5'UTR:241 | 36007 |
| N:RG203KR | 14095 |
| ORF3a:Q57H | 10929 |
| NSP2:T85I | 8451 |
| NSP6:L37F | 5507 |
| NSP12b:Y446Y | 4505 |
| ORF8:L84S | 3804 |
| ORF3a:G251V | 3792 |
| NSP4:S76S | 3743 |
| NSP15:L216L | 2479 |
| NSP14:L7L | 1813 |
| S:T723T | 1799 |
| NSP5:G15S | 1798 |
| NSP13:Y541C | 1780 |
| NSP13:P504L | 1736 |
| NSP2:P585S | 1701 |
| NSP2:I559V | 1615 |


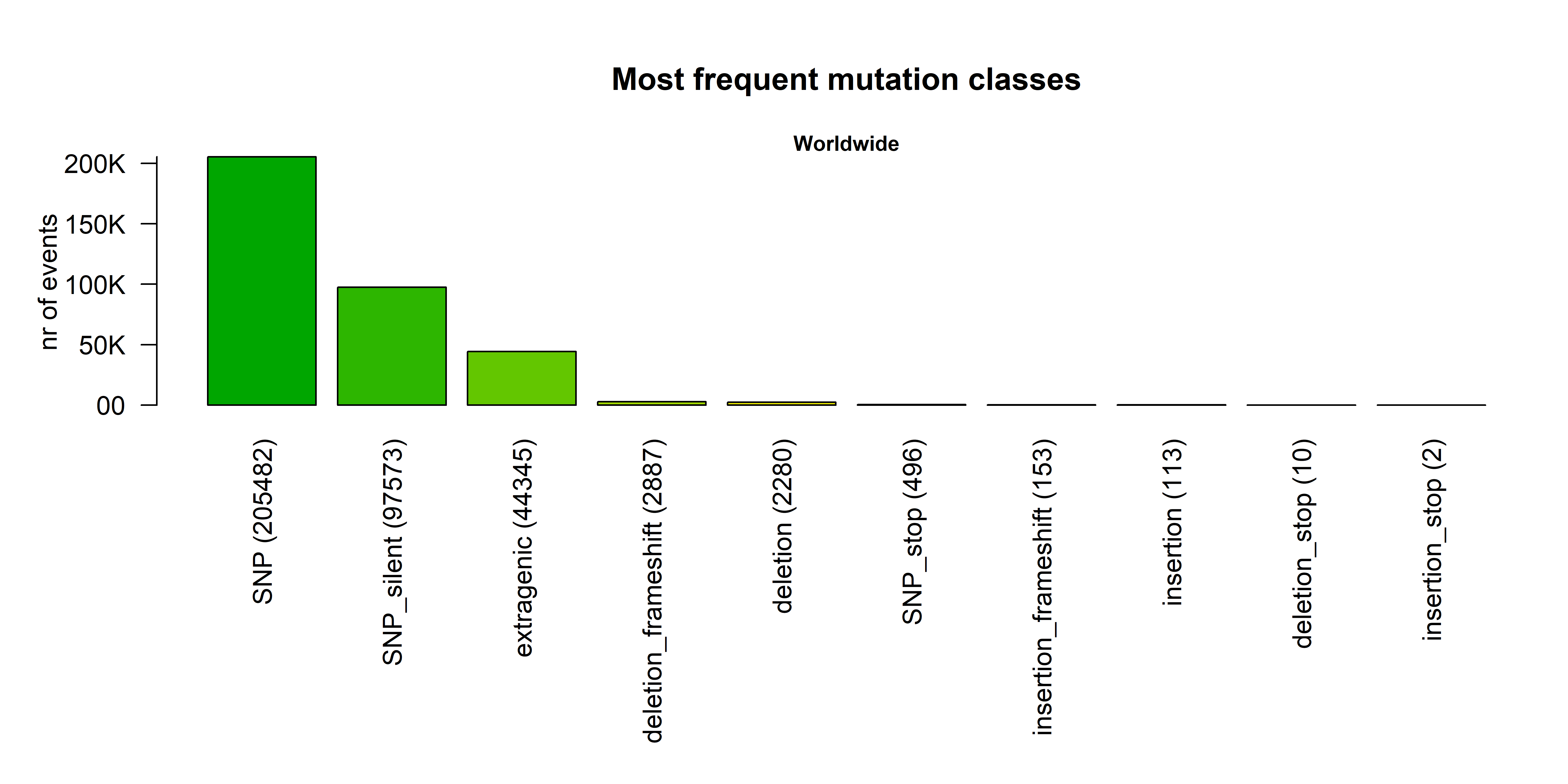


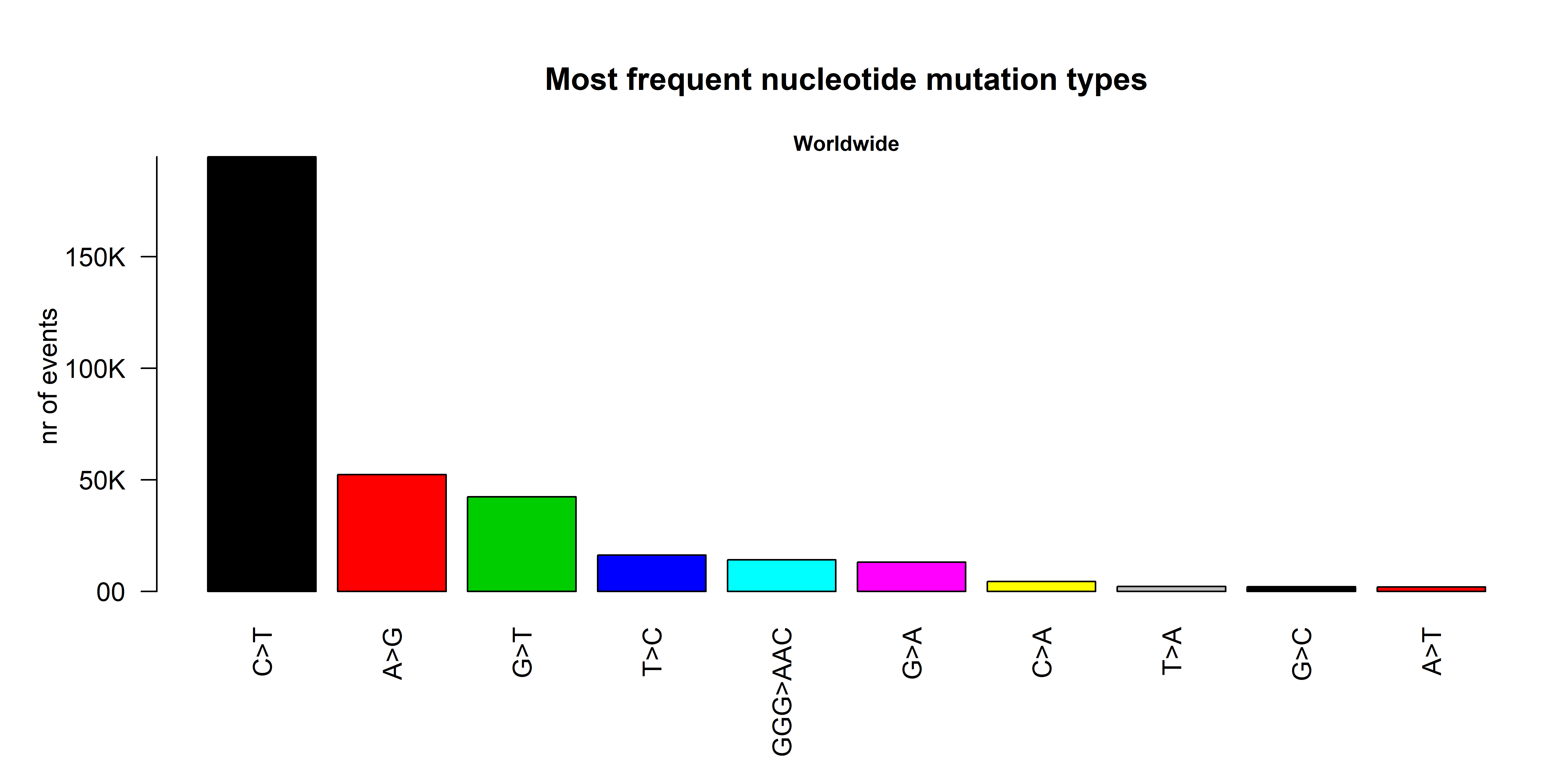


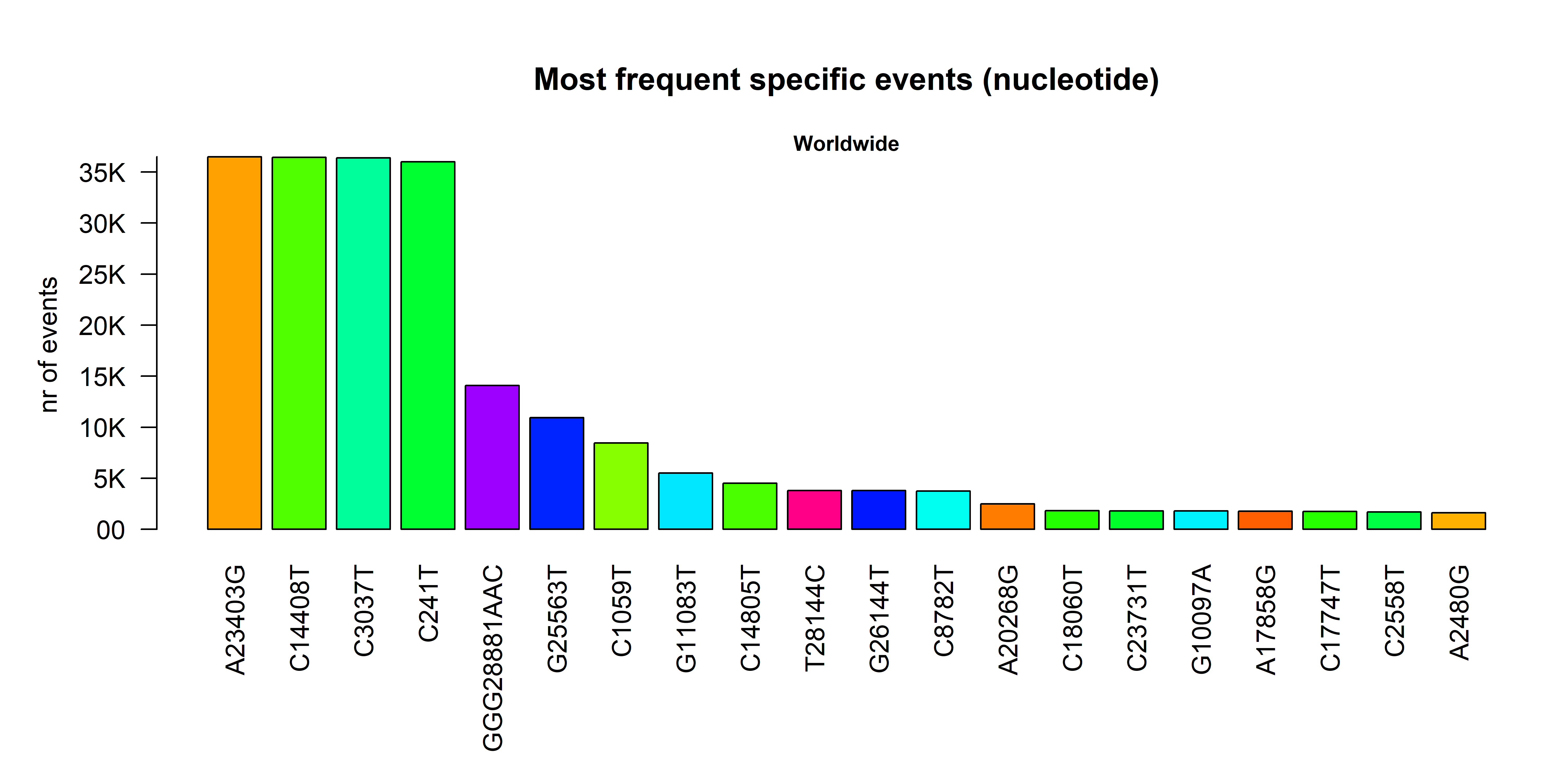

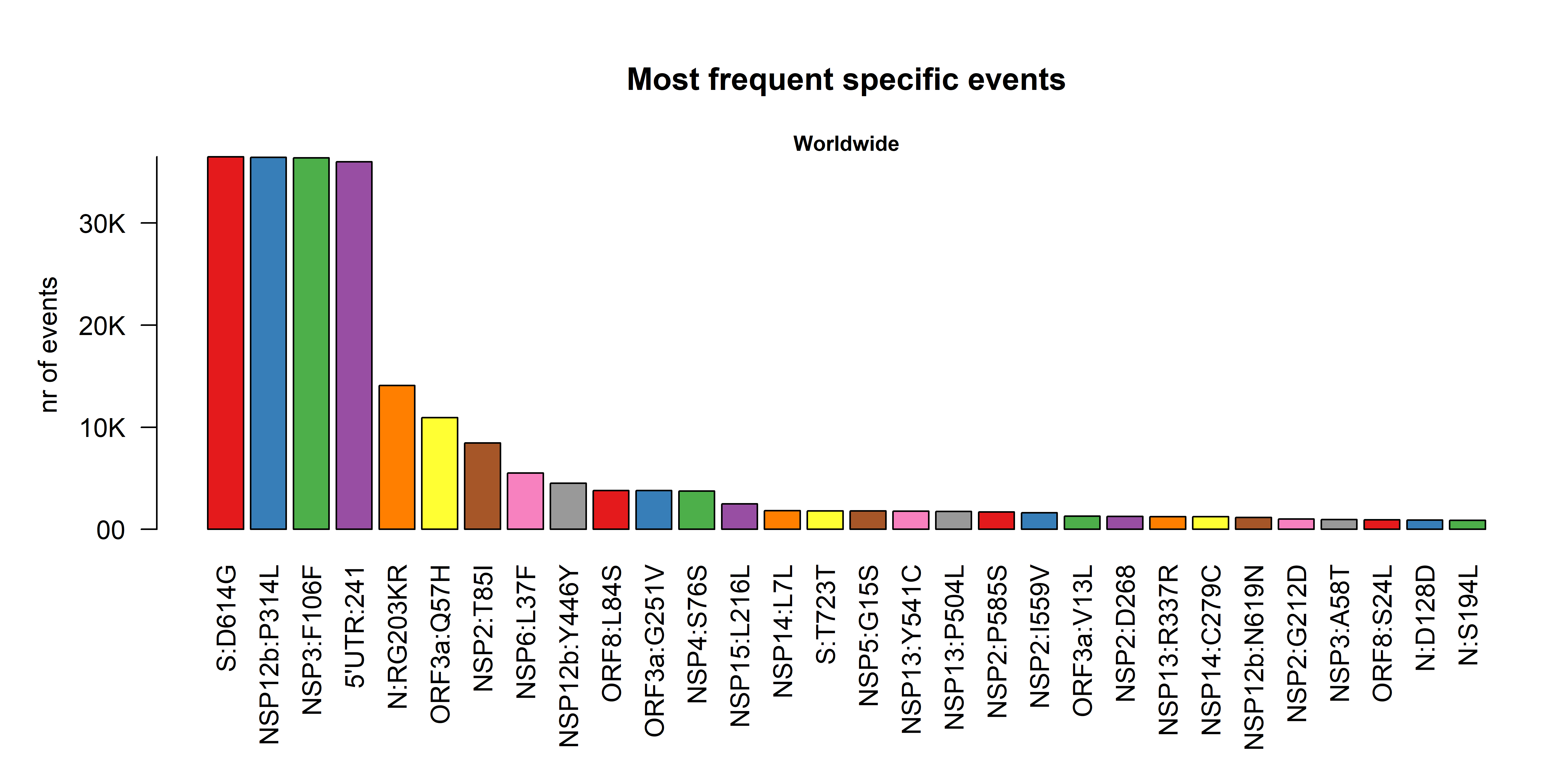

Supplement: Supplementary File 6 — Worldwide analysis of most frequent mutations categorized per class, type, nucleotide, and protein events. [file Table_6.DOCX]
