# Supplementary figures and images for: Geographic and Genomic Distribution of SARS-CoV-2 Mutations
Source: Front Microbiol. 2020 Jul 22;11:1800. doi: 10.3389/fmicb.2020.01800 (PMC7387429; doi:10.3389/fmicb.2020.01800)

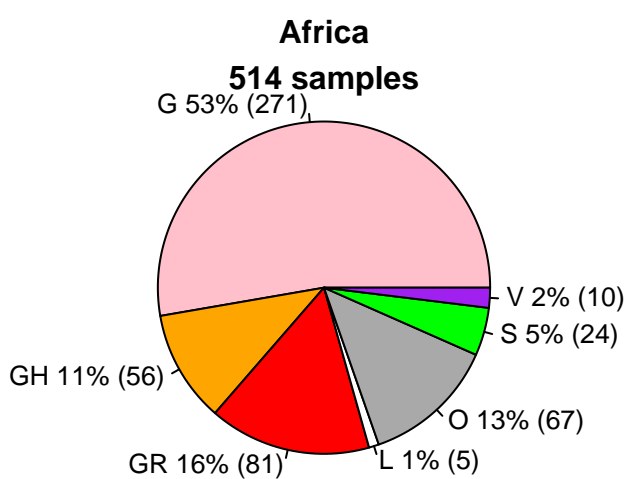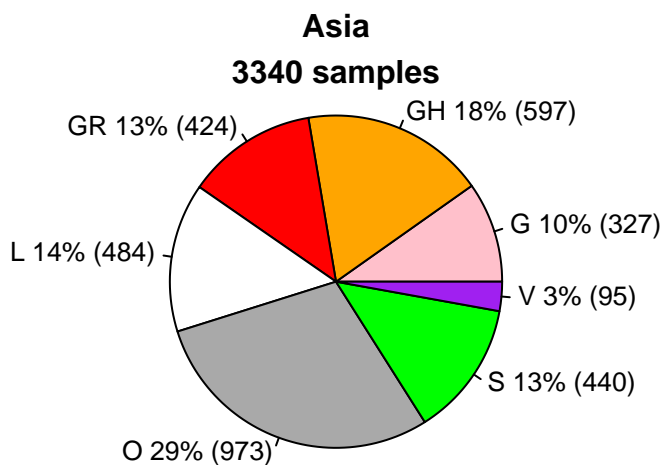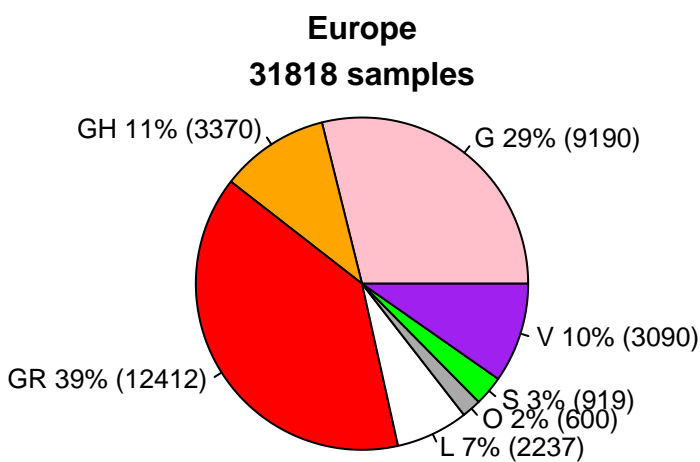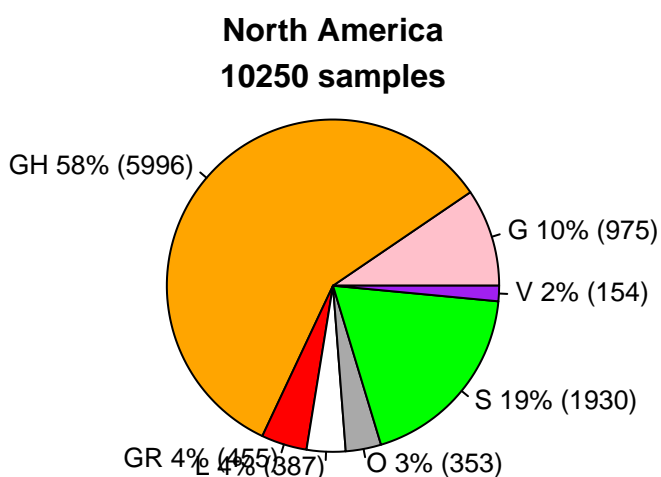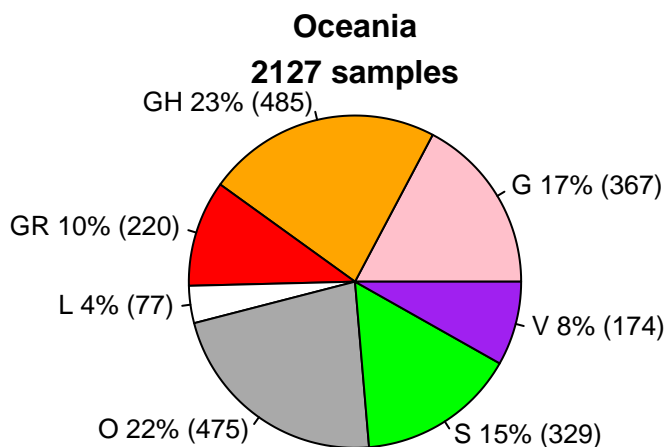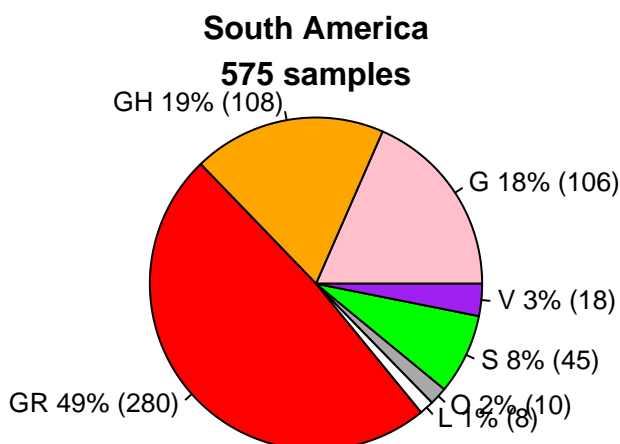

Supplement: Supplementary File 9 — Table of aa-changing mutations, categorized by protein and sorted by number of samples where the mutation has been observed. [file Data_Sheet_1.PDF]
